# A Semiautomatic Segmentation Algorithm for Extracting the Complete Structure of Acini from Synchrotron Micro-CT Images

**DOI:** 10.1155/2013/575086

**Published:** 2013-02-28

**Authors:** Luosha Xiao, Toshihiro Sera, Kenichiro Koshiyama, Shigeo Wada

**Affiliations:** ^1^Department of Mechanical Science and Bioengineering, Graduate School of Engineering Science, Osaka University, 1-3 Machikaneyama, Toyonaka, Osaka 560-8531, Japan; ^2^The Center for Advanced Medical Engineering and Informatics, Osaka University, 1-3 Machikaneyama, Toyonaka, Osaka 560-8531, Japan

## Abstract

Pulmonary acinus is the largest airway unit provided with alveoli where blood/gas exchange takes place. Understanding the complete structure of acinus is necessary to measure the pathway of gas exchange and to simulate various mechanical phenomena in the lungs. The usual manual segmentation of a complete acinus structure from their experimentally obtained images is difficult and extremely time-consuming, which hampers the statistical analysis. In this study, we develop a semiautomatic segmentation algorithm for extracting the complete structure of acinus from synchrotron micro-CT images of the closed chest of mouse lungs. The algorithm uses a combination of conventional binary image processing techniques based on the multiscale and hierarchical nature of lung structures. Specifically, larger structures are removed, while smaller structures are isolated from the image by repeatedly applying erosion and dilation operators in order, adjusting the parameter referencing to previously obtained morphometric data. A cluster of isolated acini belonging to the same terminal bronchiole is obtained without floating voxels. The extracted acinar models above 98% agree well with those extracted manually. The run time is drastically shortened compared with manual methods. These findings suggest that our method may be useful for taking samples used in the statistical analysis of acinus.

## 1. Introduction

The mammalian respiratory system can be separated into two functional zones: conducting and respiratory. The conducting zone, as an airway tree, comprises abundant branching tubes originating from the trachea, dividing dichotomously into the bronchi and bronchioles, and ending in the terminal bronchioles. Between the conducting zone and the respiratory zone, there is an intermediary region called the transitional bronchiole. Functionally, the acinus is defined as the largest airway unit, which is provided with alveoli, the smallest air-filled structures in the lung where blood/gas exchange takes place. The most precise definition is that the pulmonary acinus comprises the branched complex of alveolated airways that are connected to the same first order respiratory or transitional bronchiole [1]. Exploring the depth of the lung, for example, microstructure of the acinus, is significant for the characterization of the respiratory system at both the structural and functional level, in particular, from the viewpoint of biomechanics. 

The microstructure of the lung is harder to reconstruct and visualize relative to that of the conducting airways. Common X-ray CT images lack resolution for imaging microscale subjects, so the technique is unavailable to visualize fine structures of the lung [2]. Previously, silicone rubber cast models (3D) [3] and serial histological section reconstruction (2D-3D) [4, 5] have been used to visualize the structure of the lung parenchyma. These approaches can provide the morphological information of pulmonary acinus. However, both approaches have limitations when they are used to reconstruct the 3D structure of fine lung parenchyma for biomechanical simulation. Recently, advances in micro-CT [6, 7] and synchrotron micro-CT imaging [8–11] have made it possible to visualize the *in situ* lung anatomical structure in 3D with micrometer resolution. Because synchrotron radiation gives a much higher flux with a collimated X-ray beam compared with laboratory microfocus X-ray sources, the contrast of a synchrotron image is higher than that of a conventional X-ray source. Even though the required image resolution was available, the identification (segmentation) of the acinus structure in synchrotron micro-CT images was still hard to achieve because of the complexity of the porous structure and the razor-thin membrane wall.

A reconstruction method of a certain region of the lung parenchyma has been published [10], but it is not an entire functional structure. A complete structure of the acinus is necessary to measure the pathway of gas exchange and to simulate gas diffusion, tissue deformation, and air particle deposition [12–14]. Commonly, segmentation of the acinus structure is performed manually by an expert but is very tedious and time-consuming. In such situations, it is impractical to carry out the statistical analysis for investigating pulmonary morphology and function. Therefore, a segmentation technique for rapidly extracting the entire acinus structure is desired.

Here, we propose a semi-automatic segmentation algorithm for extracting pulmonary microstructures from three-dimensional synchrotron micro-CT images. Improvements of basic dilation, region growing, and erosion techniques are used to achieve extraction of various scales of airway structures, such as terminal bronchioles and acini. A cluster of acini structures belonging to a terminal bronchiole can also be obtained completely without isolated alveoli. 

This paper is organized as follows. The specimen and micro-CT image preparation are given in Section 2. In Section 3, preprocessing and extraction of terminal bronchioles and acini are explained in detail.  Section 4 describes our experimental results and includes a discussion.

## 2. Specimen and Micro-CT Images

**Figure 1:**
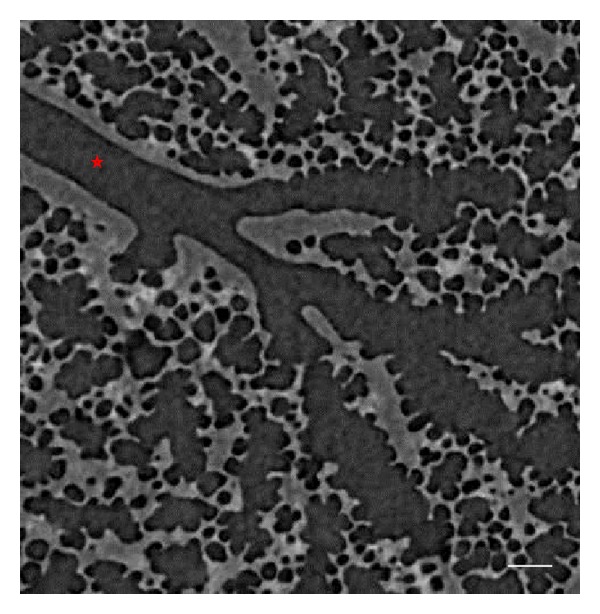
Representative synchrotron micro-CT image of the parenchyma of a mouse lung. The star marks a terminal bronchiole that is the terminal branch of conducting airways. Bar = 100 *μ*m.

The preparation of images has been previously described in detail elsewhere [9], and thus are only recapitulated briefly here. Intact, healthy mouse lungs (A/J, 9 weeks) were visualized using the synchrotron refraction-enhanced CT system at SPring-8 (http://www.spring8.or.jp/) [15]. Images were obtained when lung pressure was kept at 0 cmH_2_O. The resolution was 4000 × 4000 × 1343 voxels, with cubic voxels of 2.8 *μ*m^3^.  Figure 1 shows a representative CT image.

## 3. Segmentation Algorithm

**Figure 2:**
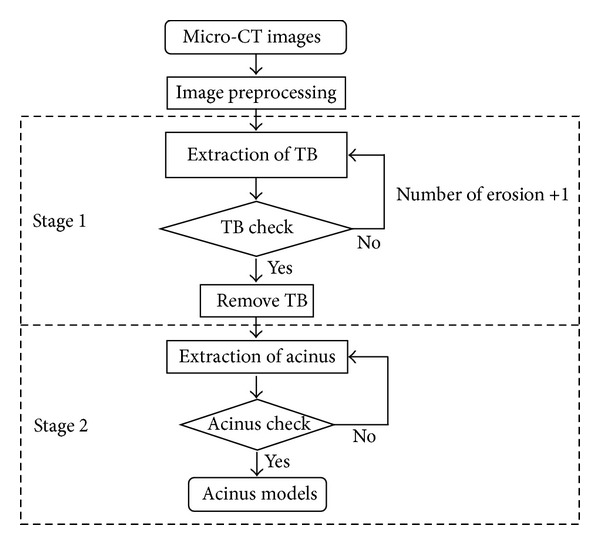
Flowchart of the semi-automatic segmentation algorithm of the pulmonary acinus. TB means terminal bronchiole.

The entire process of extracting the acinus structure from 3D synchrotron micro-CT images is shown in Figure 2. We start by preprocessing raw micro-CT data to transform grayscale images to binary and to reduce the effects of image noise on the segmentation after binarizing. Based on the hierarchical anatomical structure of the lung, with scales decreasing from bronchioles to the alveoli, the separation of connected acini was divided into two stages in terms of dimension. For clarity and simplicity, we describe this algorithm in a 2D illustration as shown in Figure 2.

### 3.1. Preprocessing: Binarization and Noise Reduction

In preprocessing, binarization and noise reduction were performed. The original micro-CT images contain too much information of lung structure to process; thus, we cropped the 3D images as 1000 × 1000 × 1000 cubes including sufficient lower lung information. As CT images are susceptible to impulse noise, and to reduce the influence of such image noise, we start by preprocessing the image data set by a simple denoising operation (medium filter) before the segmentation algorithm.

As each pixel (voxel in 3D case) must represent either air space or tissue in the lung microstructure image, all gray values were binarized into either lung tissue or air space. The selection of the threshold value for binarization can greatly influence the computational complexity of the algorithm. We used the value at the nadir of the saddle-like CT brightness histogram as an objective selection criterion for the gray scale threshold separating airspace from tissue. If the value of nadir causes irreparable noisy influence, a slight adjustment in fuzzy region can be approved reasonably. The dark gray corresponding to CT values greater than the threshold separating the two peaks was set to white (air space), and CT values less than this threshold were set to black (tissue).

**Figure 3:**
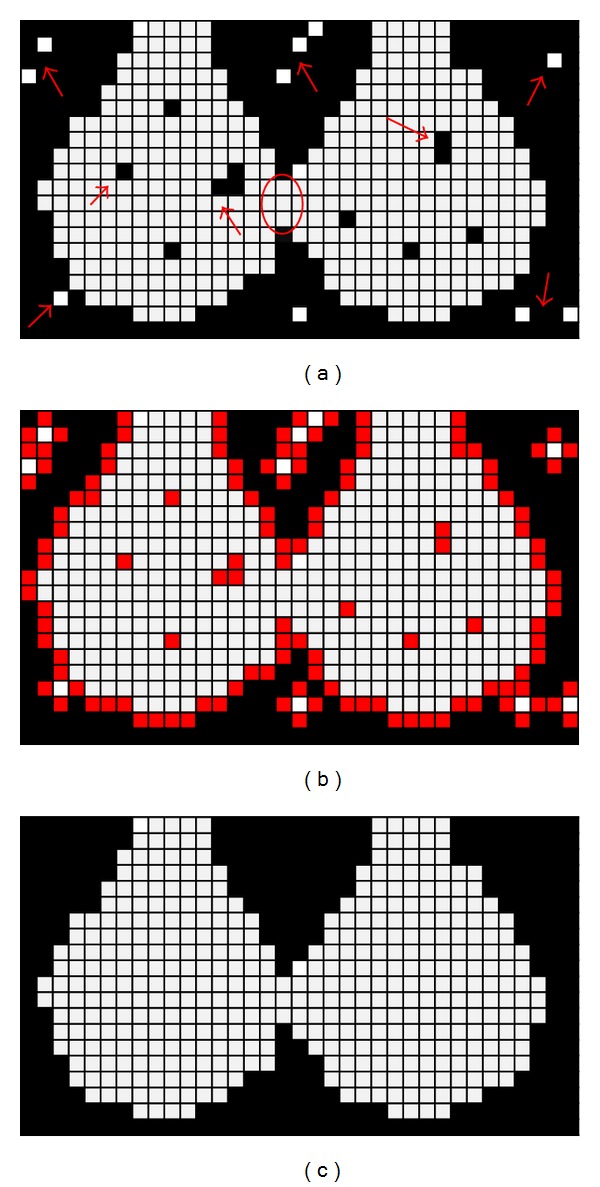
Two-dimensional illustration for preprocessing. (a) Binarization with a reasonable threshold value. (b) Elimination of the noise inside the airspace. (c) Recovery of the airspace to the original location and elimination of the outside noise.

Let us assume that two neighboring alveoli were binarized by a reasonable threshold value as in the example shown in Figure 3(a). As indicated in the figure, two types of binarized noises exist. Type I (shown in Figure 3(a) by arrows) is the imaging noise inside or outside the alveolar space, which can distort the image of the anatomic structure of pulmonary acinus, and type II (indicated in Figure 3(a) by an oval) is the image artifacts between different alveoli caused by the razor-thin wall of tissue between them. To avoid the effects of binarized noise (only type I) and to maintain the real structure of acini air space, we also need to preprocess the binarized images.

Internal noise influences the subsequent steps of preprocessing. Depending on the mean noise size, we first dilated the air space (white) by a reasonable number to fill up the internal noisy holes (Figure 3(b)). This process was performed by 6-connected erosion operator for tissue using a 3 × 3 square structuring element (in 3D, a 3 × 3 × 3 cube structuring element). Then, to eliminate the redundant voxels caused by dilation, a reasonable number of 26-connected dilation operators for tissue (several times larger than the number of erosion operator) were performed (Figure 3(c)). After this, external binarized noise is eliminated. Detailed procedures of the dilation and erosion operators are given in the Appendix.

### 3.2. Separation of Connected Acini

After reducing the type I noise from binary images, the fundamental structure of several clusters of connected acini was revealed. To some extent, type II binarized noise exists between two alveoli that belong to different acini. Thus, intact structure of a lung acinus cannot be extracted by only the region growing method [16] from a seed point in the target acinus. 

By definition, the terminal bronchiole is the previous generation of acinus structure, and the scale of it is large enough to avoid binarized noise caused by image artifacts. Thus, by deleting the terminal bronchiole from the working space, the acini belonging to it can be isolated from the entrances. However, from the entrance of each acinus, the single-seed region growing still faults to extract acinus as type II binarized noise. The problem regarding separation of connected acini translates into the elimination of type II noise between alveoli belonging to different acini.

#### 3.2.1. Extraction of Terminal Bronchioles

Using binarized 3D synchrotron micro-CT slices, the acinus was segmented starting at the terminal bronchiole. First, the extraction of the terminal bronchioles was performed by carrying out the 6-connected erosion operator using a 3 × 3 square structuring element (in 3D, a 3 × 3 × 3 cube structuring element) with a reasonable number to eliminate the connection between a terminal bronchiole and its subsequent acini. The number of erosion for extracting terminal bronchioles is based on the average path length from a pulmonary model of mouse lungs [17–19] and the resolution of a synchrotron micro-CT image. In addition, considering the nuances of airway size in different mouse stains, these parameters were adjusted slightly to increase robustness. Therefore, the equivalent erosion number was calculated as the quotient of these two parameters rounded to the nearest integer. Details of the data set are given in Table 1.

Second, to select a seed point in the remaining terminal bronchiole region, we performed single-seed region growing. The region-growing operator starts from a certain seed point inside the eroded terminal bronchiole to be segmented. The pixels neighboring this seed point were evaluated by a 3 × 3 square structuring element (in 3D, a 3 × 3 × 3 cube structuring element) to determine if they should also be considered as part of the terminal bronchiole. If so, they were added to the seed region, and the process continued as long as new pixels were added to the region. 

Finally, the 26-connected dilation operator using a 3 × 3 square structuring element (in 3D, a 3 × 3 × 3 cube structuring element), which uses the same number for erosion, was carried out to recover the terminal bronchiole to the original location. Detailed procedures of the dilation and erosion operators are given in the Appendix.

#### 3.2.2. Extraction of Acini

After deleting terminal bronchioles from the working space, all acini belonging to it can remain with isolated entrance. Because of the type II noise between alveoli belonging to different acini, an acinus still cannot be extracted directly. We continue to use the two neighboring alveoli samples as shown in Figure 3(c) and assume that they belong to two different acini.

**Figure 4:**
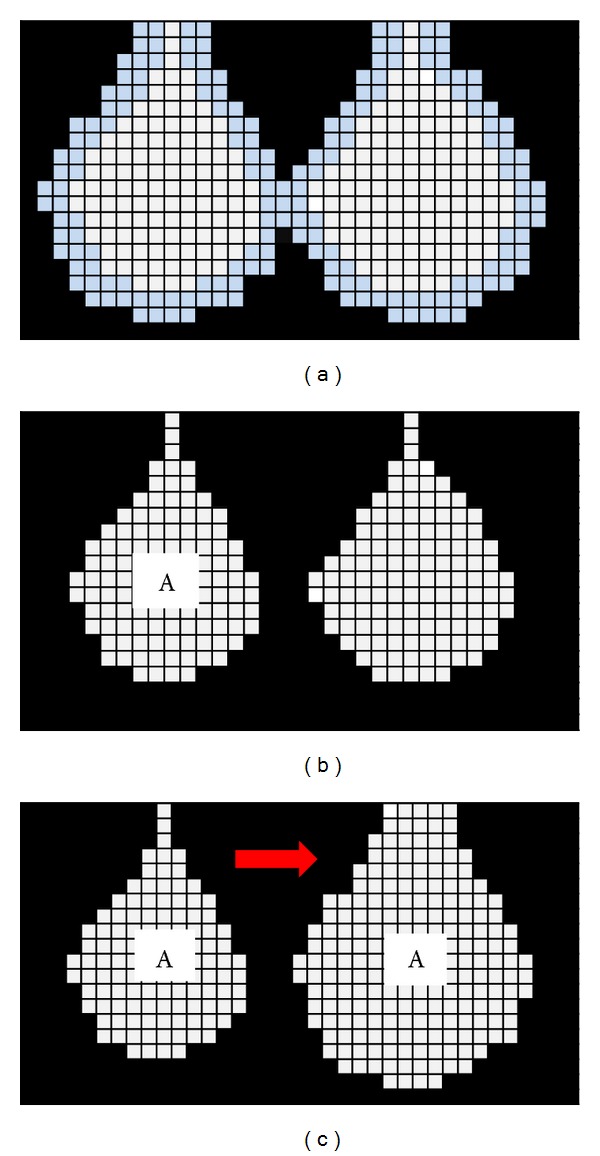
Two-dimensional illustration for the extraction of acinus. (a) Two neighbouring alveoli before erosion. (Blue shows preeroded region.) (b) Two neighbouring alveoli after erosion. (c) Dilation of the acinus to the original location.

First, a 6-connected erosion operator using a 3 × 3 square structuring element (in 3D, a 3 × 3 × 3 cube structuring element) should be performed for acinus air space. The illustration before and after erosion is shown in Figures 4(a) and 4(b). Since the dimension of acinus is very fine and the diameter of an alveolar mouth opening is usually less than the diameter of a mature alveolus, the number of erosion for extracting an acinus has a threshold. The ratio of an alveolar mouth opening to a mature alveolar diameter was assumed to be the criterion for evaluating the number of erosion for extracting the acinus structure. The threshold of the number of erosion *N* was defined as

(1) $$\begin{array}{c} N=\left\lfloor \frac{\text{Diameter}\;\text{of}\;\text{alveolar}\;\text{mouth}\;\text{opening}}{\text{Voxel}\;\text{size}\times 2}\right\rfloor . \end{array}$$

In this study, *N* = 4, where the ratio is 40% [20] and the mature alveolar diameter is 58 *μ*m [19]. 

After erosion, the target acinus was isolated from the other connected acini. A single-seed region growing operator using a 3 × 3 square structuring element (in 3D, a 3 × 3 × 3 cube structuring element) was performed to select the target acinus (assuming A in Figure 4(b)).

Finally, a 26-connected dilation operator using a 3 × 3 square structuring element (in 3D, a 3 × 3 × 3 cube structuring element), which uses the same number as erosion, was carried out to recover the target acinus to the original location (Figure 4(c)). The detailed procedures of the dilation erosion operator are given in the Appendix.

### 3.3. Validation

#### 3.3.1. Completeness

The incompleteness of the extracted acinus is caused when type II noise existing between alveoli belonging to different acini is larger than the alveolar mouth opening, that is, *N* > 4. If this phenomenon occurred, except in the extracted acinus region, the omissive parts of alveolus and the other acini remained in the working space. To obtain the omissive parts, the other acini regions were eliminated by single-seed region growing using a 3 × 3 square structuring element (in 3D, a 3 × 3 × 3 cube structuring element). Combining the extracted acinus structure and the omissive parts, a complete acinus can be restored.

#### 3.3.2. Comparison of Manual and Semi-Automatic Methods

To evaluate the accuracy of our semi-automatic segmentation algorithm, we segmented several of the same acini by our semi-automatic method and manual method. The quantitative comparison *α* was defined as match ratio

(2) $$\begin{array}{c} \alpha =\frac{n_{\text{match}}}{n_{\text{match}}+n_{\text{error}}}\times 100, \end{array}$$

where *n*_match_ is the number of matched voxels that are segmented by both methods, and *n*_error_ is the number of error voxels that are different between the two methods.

## 4. Results and Discussion

### 4.1. Original Micro-CT Image Preprocessing

**Figure 5:**
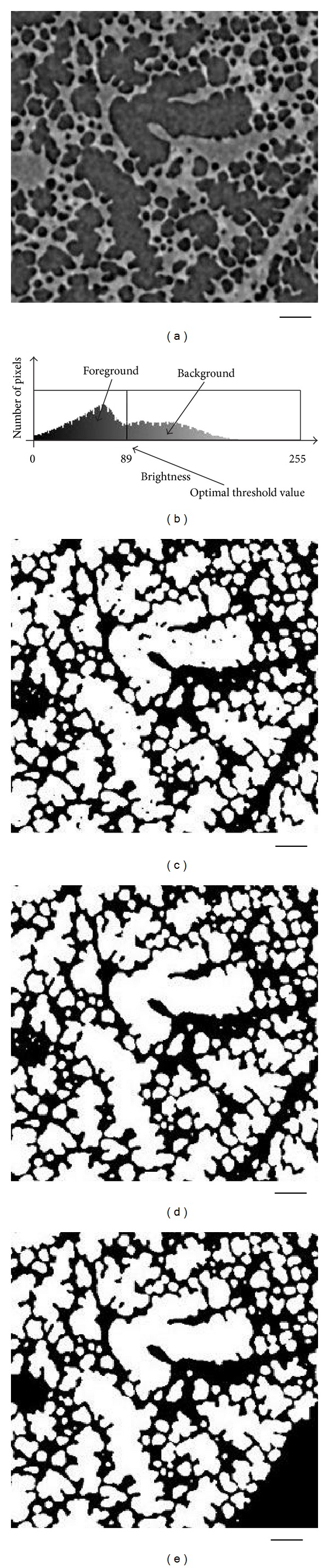
Original slice from a mouse lung synchrotron micro-CT image, binarization threshold value and noise reduction results. Bar = 100 *μ*m.

Based on the synchrotron micro-CT images, binarization and noise reduction were performed to preprocess and maintain the real acinus structure.  Figure 5 shows an example for preprocessing the original synchrotron micro-CT slice. Here, Figure 5(a) shows the original slice from a mouse lung synchrotron micro-CT image, and Figure 5(b) shows the pixel intensity histogram for binarizing the original image with an optimal threshold value. Meanwhile, the binary image, the binary image with inside noise eliminated (in air space), and the binary image with all noise eliminated are shown in Figures 5(c), 5(d), and 5(e), respectively. Based on the binarization and noise reduction of preprocessing, the images became clear, and all pixels only represent either air space (white) or tissue (black). 

It is worth pointing out that the acinus belonging to other terminal bronchioles means that such acinus was considered noise and needed to be eliminated. The black region remained in the lower right corner of Figure 5(e), which shows the elimination of acinus as noise. 

### 4.2.  3D Reconstruction of ROI

**Figure 6:**
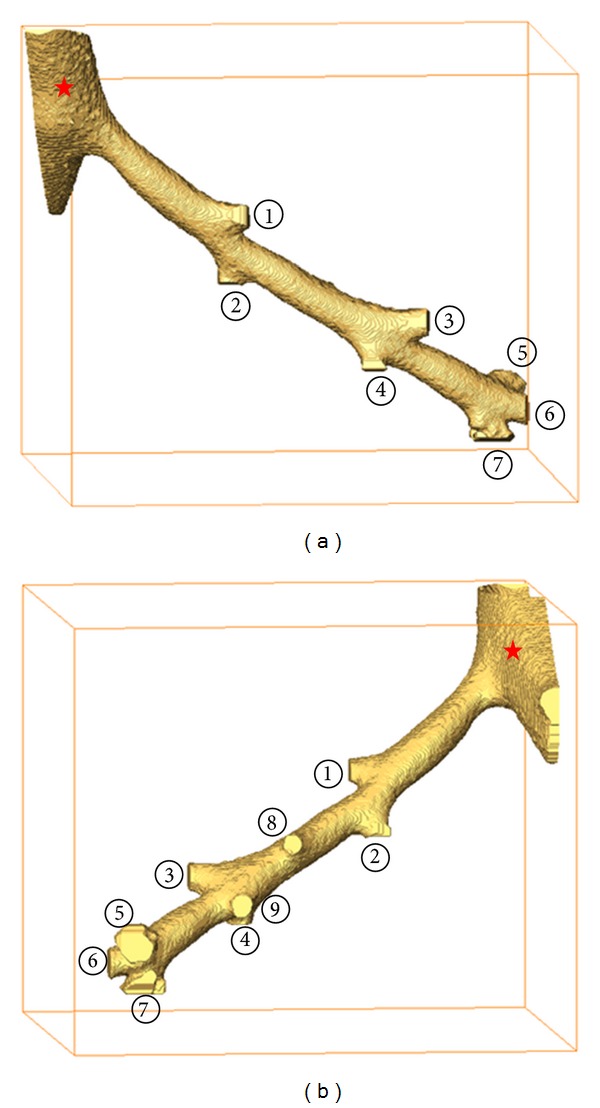
(a) 3D reconstruction of the extracted terminal bronchiole without following acini. (b) Back view of the same reconstruction data. Stars mark part of a previous generation of its associated terminal bronchiole. Entire branches are marked by serial numbers. The dimensions of the bounding boxes (a) and (b) are 1.26 × 1.06 × 1.26 mm^3^.

Using the preprocessed binary images, the acinus was segmented starting from the deletion of terminal bronchioles. A representative terminal bronchiole without the subsequent acini was segmented as shown in Figure 6. According to the branch point of acini belonging to this terminal bronchiole, 9 clusters of acini were detected and positioned for further segmenting. 

**Figure 7:**
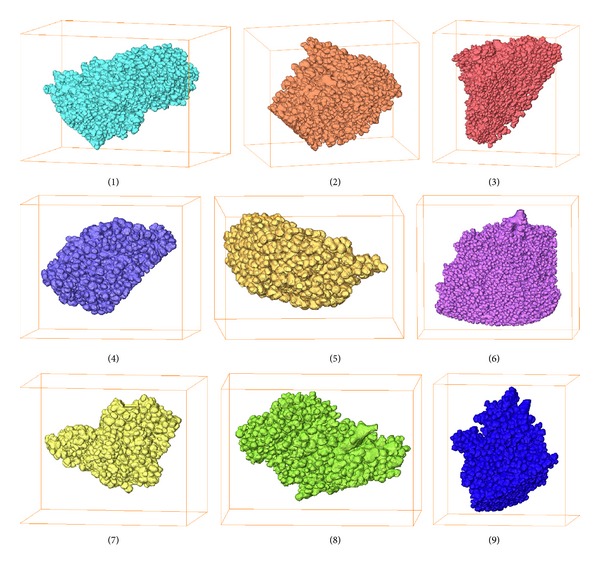
3D reconstruction of all 9 extracted acini. The dimensions of the bounding boxes (mm^3^): (1) 1.23 × 1.20 × 1.68; (2) 0.98 × 1.06 × 1.37; (3) 1.37 × 0.95 × 1.34; (4) 0.81 × 0.87 × 1.06; (5) 0.56 × 0.84 × 0.81; (6) 1.46 × 1.68 × 0.98; (7) 0.81 × 0.84 × 1.04; (8) 0.81 × 0.78 × 1.15; and (9) 0.76 × 0.95 × 1.01.

Using our semi-automatic segmentation algorithm, acinus structures were segmented quickly. The extracted acini are shown in Figure 7. All these models, except number 5, were deemed complete depending on the threshold *N*. The branches of the terminal bronchiole correspond to the entrances of each acinus. 

**Figure 8:**
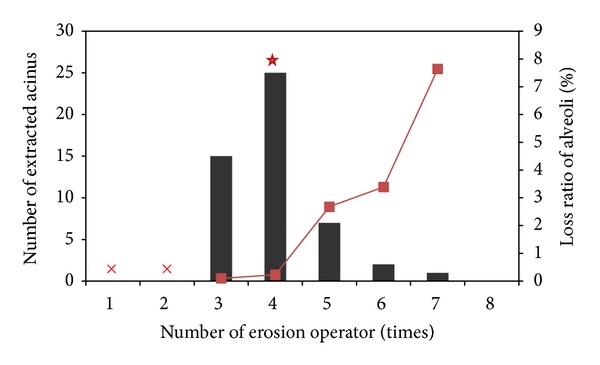
Total number of samples is 50. ×  marks the number of erosion operations that are not suitable for extracting an acinar model, and the red star marks the ideal number of erosion operations. Columns show the number of extracted acini (left *Y* axis) and the line shows the loss ratio of alveoli (right *Y* axis).

To verify the threshold of completeness criterion, a mass of experiments were carried out via trial and error. We extracted 50 intact acinus samples to create a database. The distribution of the number of erosion for acinus is shown in Figure 8. The red star marks the ideal value of the number of erosion as the high ratio of extraction and low ratio of loss of alveolar fine texture, which agrees well with the threshold *N*. When the number of erosion is less than or equal to 2, type II noise between neighbouring alveoli belonging to different acini is too large to eliminate. Therefore, the initial number of erosion for all segmentation process is set as 3. 

As indicated in Figure 8, when the number of erosion is larger than 4, the loss ratio of alveoli increases from 0.23 to 7.64. Even though the percentage is not very large, the omission of alveolar structure influences the morphometry such as an investigation of an airway branch to the terminal alveolus.

**Figure 9:**
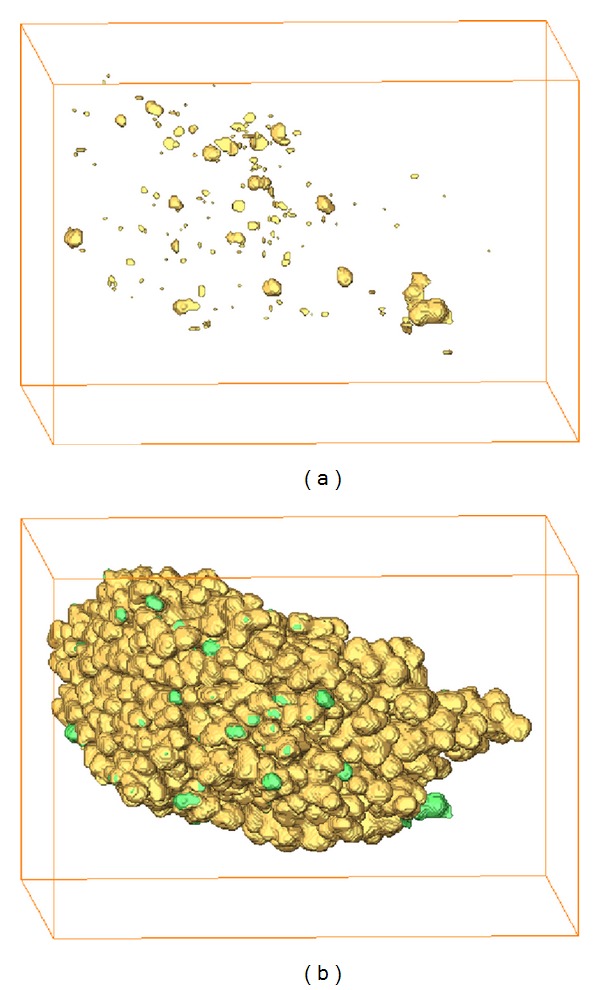
3D reconstruction of the adjustment for an incomplete acinus. (a) Omission of alveoli fine texture. (b) Repaired complete acinus.

The adjustment of an incomplete acinus was shown by 3D reconstruction of the number 5 acinus in Figure 9. This incomplete acinus was extracted by five erosion operations, which is larger than the threshold of completeness check. The omission of alveoli fine texture after segmentation and the recovered result are shown in Figures 9(a) and 9(b). 

**Figure 10:**
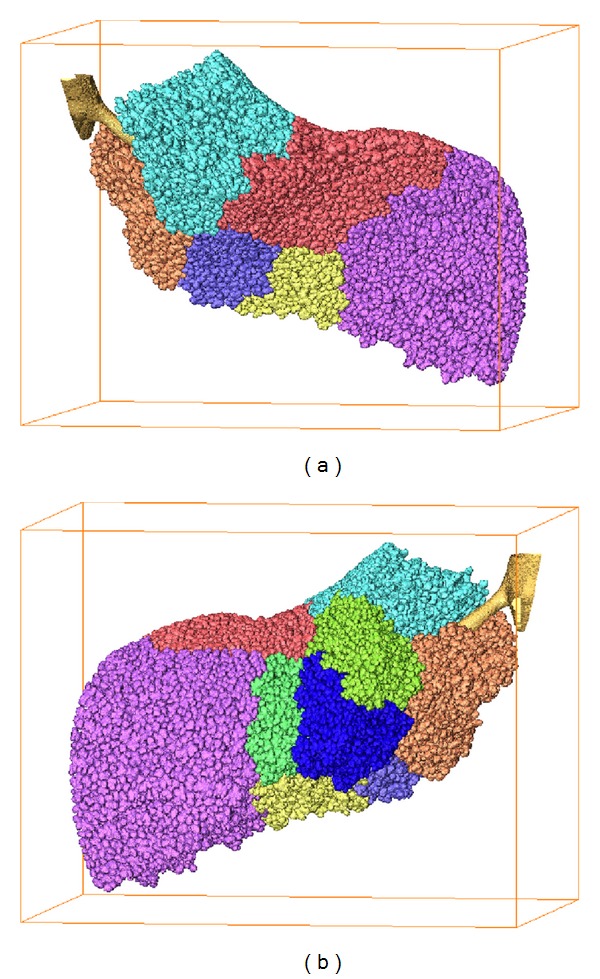
(a) 3D reconstruction of the extracted terminal bronchiole acini. (b) Back view of the same reconstruction data. The dimensions of bounding boxes (a) and (b) are 2.80 × 2.18 × 2.21 mm^3^.

Here, we successfully segmented and repaired nine clusters of acini belonging to the same terminal bronchiole. All the acini fill the space in the lung where they localized as shown in Figure 10. 

### 4.3. Comparison of Manual and Semiautomatic Results

Both qualitative and quantitative comparisons for the manual and semi-automatic methods were performed. Four acini models were extracted manually by commercial software (Amira 5.4.1), and the same regions were also extracted by our semiautomatic algorithm. Figure 10 shows the 3D reconstruction of these four acini models. Intuitively, the results of the segmentation by the two methods were basically the same except for the entrance. 

**Figure 11:**
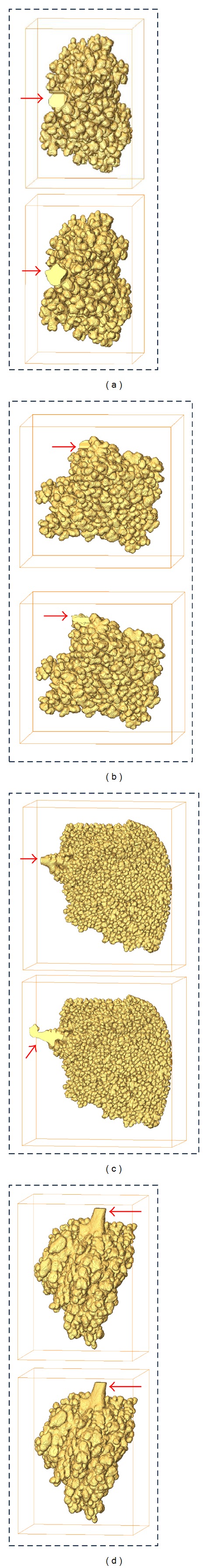
Four intact reconstructed acini. In each group, the upper acinus was extracted by our semi-automatic method, and the lower acinus was extracted by the manual method.

As indicated in the (b) and (c) group in Figure 11, the sharpness of the acinar entrance is significantly different. The manual method has difficulty processing an acinus that has a direction of entrance perpendicular to the direction of the 3D image slice. The processing time of an acinus model running manually was about 1 wk; otherwise, the running time of the semiautomatic algorithm was about 2 hr.

Table 2 shows the quantitative comparison *α*, and overall values are above 98%.

## 5. Conclusion 

We have described a semi-automatic segmentation algorithm for extracting lung microstructure acinus from synchrotron micro-CT images. The algorithm uses a combination of binary image processing operators: erosion, region growing, and dilation based on the multiscale structure of the lung. A cluster of extracted lung acini structures belonging to the same terminal bronchiole is possible to fill in the space in the lung where it is localized. The extracted acini models with *α* values above 98% agree well with those extracted manually. The run time is drastically shortened compared with the manual method.

## Figures and Tables

**Figure 12:**
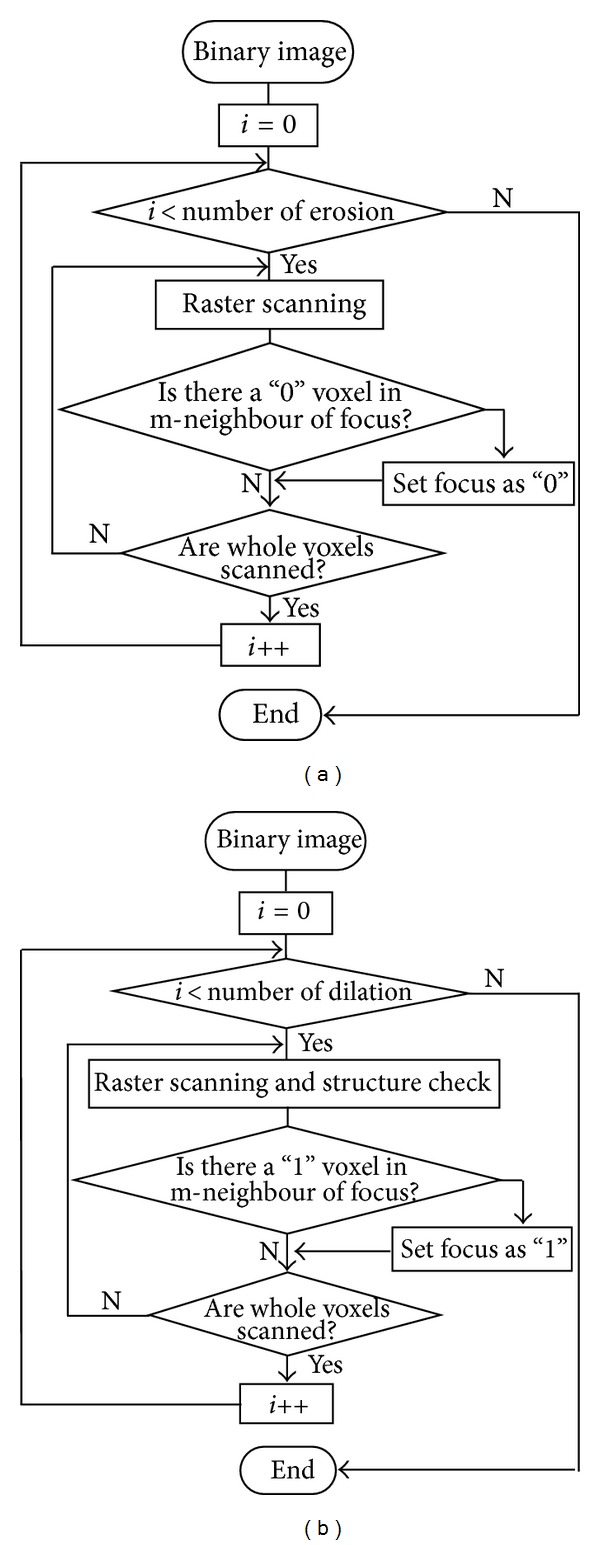
Flowchart of the modified erosion (a) and dilation (b) operator (for air space).

**Table 1:** Diameter of the small airways and equivalent number of erosion for elimination.

Airway name	Diameter (*μ*m)*	Erosion number for eliminating airway
Terminal bronchiole	160	29 times
Transitional and respiratory bronchiole	100	18 times
Alveolar duct	90	15 times
Alveolus	58	11 times

*Taken from previous morphometric data published by Oldham and Robinson [17] on *in situ* lung casts of BALB/c mice. The lung pressure is the same as in this study.

**Table 2:** Quantitative comparison of the four pairs of acinus models in Figure 11.

Sample No.	n_match_	*n*_error_	*α*

(a)	1648004	7537	99.5
(b)	2418765	46274	98.1
(c)	11718520	9990	99.9
(d)	4234352	714	99.9

## Appendix

### Detailed Procedure of Image ****Processing Operator

Figure 12 shows the flowchart of erosion (a) and modified dilation (b) operator (for air space) used in our algorithm. The erosion operator is the basic effect of the operator, which is to erode away the boundaries of regions of foreground voxels. The dilation operator is another basic effect of the operator which is to gradually enlarge the boundaries of regions of foreground voxels. In particular, the dilation operator was modified on the basis of an acinus structure.

For air space, “1” means foreground and “0” means background, otherwise for tissue, “0” means foreground and “1” means background; *m* is the number of connectedness.

## References

[1] Weibel ER, Sapoval B, Filoche M (2005). Design of peripheral airways for efficient gas exchange. *Respiratory Physiology and Neurobiology*.

[2] Aykac D, Huffman EA, McLennan G, Reinhardt JM (2003). Segmentation and analysis of the human airway tree from three-dimensional X-ray CT images. *IEEE Transactions on Medical Imaging*.

[3] Haefeli-Bleuer B, Weibel ER (1988). Morphometry of the human pulmonary acinus. *Anatomical Record*.

[4] Bal HS, Ghoshal NG (1988). Morphology of the terminal bronchiolar region of common laboratory mammals. *Laboratory Animals*.

[5] Mercer RR, Crapo JD (1987). Three-dimensional reconstruction of the rat acinus. *Journal of Applied Physiology*.

[6] Litzlbauer HD, Neuhaeuser C, Moell A (2006). Three-dimensional imaging and morphometric analysis of alveolar tissue from microfocal X-ray-computed tomography. *American Journal of Physiology*.

[7] Parameswaran H, Bartolák-Suki E, Hamakawa H, Majumdar A, Allen PG, Suki B (2009). Three-dimensional measurement of alveolar airspace volumes in normal and emphysematous lungs using micro-CT. *Journal of Applied Physiology*.

[8] Sera T, Uesugi K, Yagi N (2005). Localized morphometric deformations of small airways and alveoli in intact mouse lungs under quasi-static inflation. *Respiratory Physiology and Neurobiology*.

[9] Sera T, Yokota H, Tanaka G, Uesugi K, Yagi N, Schroter R The relationship between alveoli and alveolar ducts in lung inflation.

[10] Tsuda A, Filipovic N, Haberthür D (2008). Finite element 3D reconstruction of the pulmonary acinus imaged by synchrotron X-ray tomography. *Journal of Applied Physiology*.

[11] Litzlbauer HD, Korbel K, Kline TL (2010). Synchrotron-based micro-CT imaging of the human lung acinus. *Anatomical Record*.

[12] Sera T, Uesugi K, Yagi N (2005). Localized morphometric deformations of small airways and alveoli in intact mouse lungs under quasi-static inflation. *Respiratory Physiology and Neurobiology*.

[13] Schroter RC, Sudlow MF (1969). Flow patterns in models of the human bronchial airways. *Respiration Physiology*.

[14] Tsuda A, Henry FS, Butler JP (2008). Gas and aerosol mixing in the acinus. *Respiratory Physiology and Neurobiology*.

[15] Goto S, Takeshita K, Suzuki Y (2001). Construction and commissioning of a 215-m-long beamline at SPring-8. *Nuclear Instruments and Methods in Physics Research A*.

[16] Toriwaki J, Murakami S (2010). *Introduction to 3D Image Processing*.

[17] Oldham MJ, Robinson RJ (2007). Predicted tracheobronchial and pulmonary deposition in a murine asthma model. *Anatomical Record*.

[18] Oldham MJ, Phalen RF (2002). Dosimetry implications of upper tracheobronchial airway anatomy in two mouse varieties. *The Anatomical Record*.

[19] Mercer RR, Russell ML, Crapo JD (1994). Alveolar septal structure in different species. *Journal of Applied Physiology*.

[20] Mercer RR, Laco JM, Crapo JD (1987). Three-dimensional reconstruction of alveoli in the rat lung for pressure-volume relationships. *Journal of Applied Physiology*.

